# Development of Cardiovascular Indices of Acute Pain Responding in Infants: A Systematic Review

**DOI:** 10.1155/2016/8458696

**Published:** 2016-04-20

**Authors:** Jordana A. Waxman, Rebecca R. Pillai Riddell, Paula Tablon, Louis A. Schmidt, Angelina Pinhasov

**Affiliations:** ^1^Department of Psychology, York University, Toronto, ON, Canada; ^2^Hospital for Sick Children, Toronto, ON, Canada; ^3^Department of Psychiatry, University of Toronto, Toronto, ON, Canada; ^4^Department of Psychology, Neuroscience, and Behaviour, McMaster University, Hamilton, ON, Canada

## Abstract

*Background*. Cardiovascular indices of pain are pervasive in the hospital setting. However, no prospective research has examined the development of cardiac responses to acutely painful procedures in the first year of life.* Objectives*. Our main goal was to synthesize existing evidence regarding the development of cardiovascular responses to acutely painful medical procedures over the first year of life in preterm and term born infants.* Methods*. A systematic search retrieved 6994 articles to review against inclusion criteria. A total of 41 studies were included in the review.* Results*. In response to acutely painful procedures, most infants had an increase in mean heart rate (HR) that varied in magnitude both across and within gestational and postnatal ages. Research in the area of HR variability has been inconsistent, limiting conclusions.* Conclusions*. Longitudinal research is needed to further understand the inherent variability of cardiovascular pain responses across and within gestational and postnatal ages and the causes for the variability.

## 1. Introduction

Although skepticism towards infant pain characterized much of the 20th century research and clinical practices [1], it is now well established that infants' pain transmission pathways in the brain are fully developed by 22 to 24 weeks of gestation [2]. Conversely, pain inhibitory pathways are not fully developed in infants, suggesting that infants may feel even more pain than older children [2]. Improper management of infant acute pain has been associated with various short- and long-term negative physiological and psychological consequences. Specifically, increased metabolic rate during painful experiences has been associated with short-term consequences such as exacerbating injury, increased potential for chronic pain, delayed wound healing, increased risk of infection, and alterations in pain sensitivity [3–5]. Additionally, long-lasting consequences include delays in motor and brain development, as well as deficits in cognition and emotion regulation [6–11]. Therefore, it is important to establish empirically based behavioural and physiological pain assessment tools that can be utilized in infancy to begin the pain management process.

The major challenge with infant pain assessment is that neonates cannot self-report their subjective experience of pain. Moreover, there is a lack of agreement on the best proxy modality of assessing infant pain, whether it is cortical, biochemical, physiological, or behavioural [12]. Moreover, recent work has suggested discordance not only among modalities [13, 14], but also within an assessment modality [15]. For example, the validity and reliability of physiological measures of infant pain are presently disputed, due to the fact that these measures are influenced by additional variables or covariates that have not been properly been taken into account (e.g., infection and respiratory rate) [16].

Despite the above-mentioned disputes, cardiophysiological indices of pain, such as heart rate (HR) and HR variability (HRV), are pervasive in the hospital setting [17]. Indeed, cardiac measures are well-established noninvasive proxies of cardiac autonomic control and have been integrated in well-established pain assessment tools for preterm and term born infants, as well as young children [3, 18–23]. However, despite this integration into mainstream clinical practice, there appears to be no research that has systematically examined the developmental differences of cardiac responses to acutely painful procedures in either preterm or term born infants longitudinally. Systematic research with a behavioural indicator of pain has suggested extreme variability across the first year of life [15] and given the established differences in nervous system processing between preterm and full term born infants [24–27], it behooves researchers to more systematically examine cardiac responding to pain and its validity as a proxy for pain perception [28, 29]. The purpose of this systematic review is to synthesize existing evidence on the development of cardiovascular responses to acutely painful medical procedures over the first year of life in both preterm and term born infants. Studies will be organized first according to the age of the infant at measurement, then subdivided by the gestational age at birth, and then further subdivided by the type of cardiac measurement.

## 2. Method

### 2.1. Search Strategy

With the assistance of an academic librarian at the University of Toronto, a systematic search was conducted in Medline, Embase, PsychINFO, and CINAHL in July 2014 for English-language references. Searches were limited to articles published from 1970 to 2014 in order to encompass historical and contemporary articles and reviews. Search terms related to acute pain procedures, cardiovascular measures, and infants (0–3 years of age) were systematically paired (see Supplementary File 1 for Medline search in Supplementary Material available online at http://dx.doi.org/10.1155/2016/8458696). We also hand searched reference lists of relevant studies and systematic reviews on cardiovascular responses to acute pain in infants. Our review followed an a priori protocol according to the Preferred Reporting Items for Systematic Reviews and Meta-Analyses (PRISMA) guidelines [30]. The review protocol was registered on the PROSPERO website before data extraction (registration number CRD42015016398) [7].

### 2.2. Inclusion and Exclusion Criteria and Study Selection

We included prospective observational or descriptive studies of individuals equal to or under 3 years of age undergoing an acutely painful procedure, which was monitored using a cardiovascular measure. Our definition of observational studies included cohort studies in which participants were prospectively identified and followed up during acutely painful procedures using cardiovascular indices, as well as cross-sectional studies that observed an acutely painful procedure using a cardiovascular measure across different gestational or postnatal ages. We also included control group data from pain manipulation studies and prospective randomized or randomized controlled trials (RCTs) that investigated the effectiveness of pain management strategies using cardiovascular measures.

Studies were excluded if they described nonhuman animal models of pain, did not measure an acutely painful event nor include a cardiovascular measure of acute pain, were prospective randomized, RCTs, or pain manipulations that did not include a control group, were review articles, case studies, or conference abstracts, or studies that included participants that differed in age at measurement (i.e., collapsing over one or more months), or gestational age (GA) (i.e., collapsing across at least four months of GA). Of note, most studies that were discarded for collapsing over age of measurement were averaging over age spans within infancy greater than 6 months.

**Figure 1:**
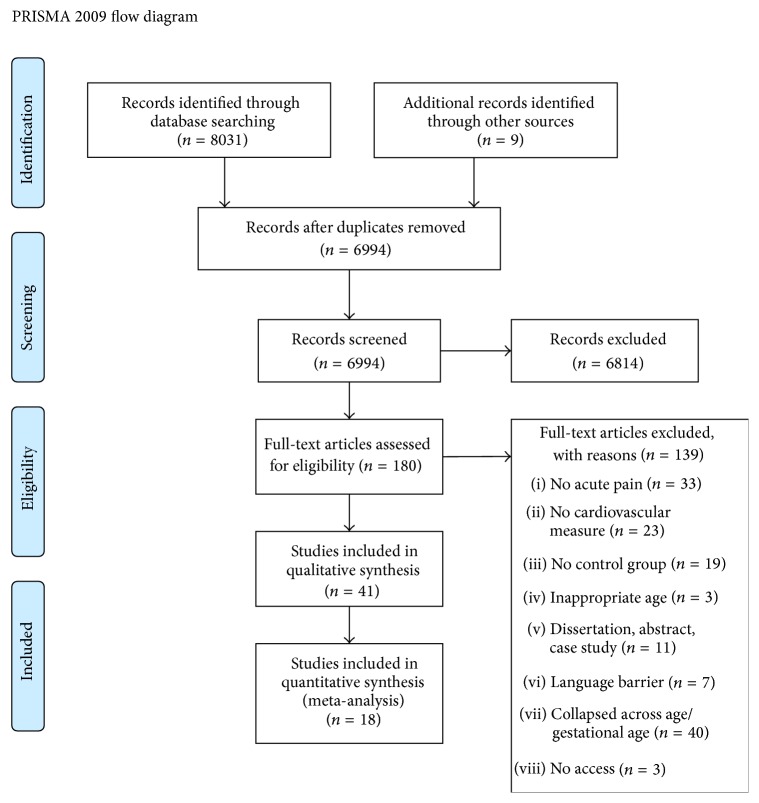
Included study flow chart following PRISMA guidelines.

Two authors designed the abstract selection criteria with an initial selection of 500 abstracts (Jordana A. Waxman and Rebecca R. Pillai Riddell). Three authors (Angelina Pinhasov, Jordana A. Waxman, and Paula Tablon) independently read and selected from all the retrieved references and abstracts. Any disagreements between reviewers were resolved through discussion. The percent agreement between the raters ranged from 0.96 to 1.0. Full texts of potentially eligible studies were retrieved (see Figure 1).

### 2.3. Data Extraction and Quality Assessment

A database was created recording GA at birth, postnatal age at measurement, a description of the cardiovascular results, and any covariates that were included when analyzing whether there were differences in cardiovascular measures following an acutely painful medical procedure. It was important to investigate covariates included in the studies, as there are a number of physiological and behavioural variables known to affect the cardiovascular system [16]. We reasoned that delving into what variables were controlled for might help explain why there is variability in cardiovascular measures. Where information was incomplete, the authors were contacted by email.

Due to the fact that a gold-standard quality assessment measure was not available for observational studies [31], a modified checklist combining Downs and Black [32] and Crombie and McQuay [33] was utilized (see Supplementary File 2 for the checklist). These measures were chosen based on a multidisciplinary collaborative review in the field discussing quality in case-control, cohort, and cross-sectional studies [31]. Fifty percent of the extractions were consensus coded for quality scores to ensure reliability. Disagreements were minimal and were resolved through discussion to obtain a final score for each paper. Criteria were scored as “Yes” (1), “No” (0), or “Unable to Determine.” Positively scored criteria were added to obtain a total quality score for the paper. The maximum obtainable score was 20 for cross-sectional studies and 21 for cohort studies. The results were expressed as percentages of the total obtainable score.

### 2.4. Analysis

We aimed to synthesize evidence on the development of cardiovascular responses to acutely painful procedures in preterm and term born infants. For qualitative analysis, group-specific data were first separated by age at measurement and subsequently subdivided by GA at birth, as well as cardiovascular outcome measures (i.e., mean heart rate (HR), HR change, maximum HR, total heart rate variability (total HRV), low frequency heart rate variability (LF HRV), high frequency heart rate variability (HF HRV), and low frequency/high frequency ratio (LF/HF ratio)).

## 3. Results

### 3.1. Studies Included

We identified 6994 articles from the electronic searches after removal of duplicates. These articles were then reviewed by title and abstract and were included or excluded based on a priori selection criteria. A total of 180 articles were then reviewed by full-text review, and of these, 41 articles (involving 1552 participants) fulfilled the inclusion criteria [24–27, 34–71]. These studies underwent quality assessment and data extraction and were included in the final review.

### 3.2. Study Characteristics

Table 1 provides a detailed overview of the studies included, including sample size, country of origin, GA at birth, postnatal age at measurement, acutely painful procedure, cardiovascular measure, study design, and quality assessment score.

Generally speaking, a quarter of the studies were from Canada, a quarter from the United States, and a quarter from Europe, with the remaining studies coming from Asia, the Middle East, and Brazil. The majority of studies were randomized trials and encompassed infants born between 24 and 42 weeks GA that were tested between postnatal day 1 and postnatal month 4. The most common acutely painful procedure that was utilized in the studies was heel stick, and mean HR was the most frequently used cardiovascular measure. In terms of the range of quality scores for the papers, the lowest score was 40% [27], the median quality score for the papers was 75%, and the highest score was 86% [69].

Age categorizations were difficult to obtain due to the variability between studies in the age groups they analyzed. Based on the available data, the results will be organized by the following postnatal ages (i.e., age at measurement): 7 postnatal days or less, 1 to 2 postnatal weeks, 3 postnatal weeks, and 1, 2, 3, and 4 postnatal months. Since the majority of data are published on infants within the first 7 postnatal days, tables will only be presented for these studies (see Tables 3 [Table tab4] [Table tab5] [Table tab6] [Table tab7] [Table tab8]–9). In addition, within each age at measurement category, results will then be subdivided by accepted categorizations of GAs [72] and the cardiovascular measures examined. Due to the large variability in choice of covariates only the presence of covariates will be noted, with a comprehensive list being provided in Table 2 across studies.

#### 3.2.1. Age at Measurement: Seven Postnatal Days or Less

*Extremely Preterm: 25 to 27 Weeks of GA*

*Mean Heart Rate*. One high quality study used mean HR to describe the acute pain experience following a heel stick in those born at 25–27 weeks of GA and were measured at 5.50 postnatal days [24]. The authors found that those born at 25–27 weeks of GA did not have a significant increase in mean HR in response to a heel stick in the first week of life. The study found that the mean HR was 172.38 bpm in response to heel stick.

*Very Preterm: 28 to 32 Weeks of GA*

*Mean Heart Rate*. A total of 3 studies investigated the mean HR response to heel stick in those born at 28 to 32 weeks of GA and measured at 3 to 6 postnatal days [24, 39, 56]. The three studies found that HR significantly increased following the heel stick [24, 39, 56]. The studies found that mean HR postacute pain ranged from 155.25 to 169.27 bpm (see Table 3). The variability may be due to only one study including covariates (i.e., number of prior heel sticks, duration of blood draws, sex, and baseline HR) in their analysis of the cardiovascular measures [73]. Overall, the studies were generally of high quality.

*Heart Rate Change*. One lower quality study examined mean HR change in response to heel stick at 4 postnatal days [44] and found that HR was significantly higher during blood collection compared to baseline HR (see Table 4).

*Heart Rate Variability*. One high quality study investigated LF and HF HRV, as well as the LF/HF ratio in response to heel stick at 6 postnatal days [39]. The authors found that LF and HF HRV increased in response to heel stick (see Tables 5 and 6, resp.), while the LF/HF ratio decreased in response to heel stick (see Table 7).

*Moderate Preterm: 32 to 34 Weeks GA*

*Mean Heart Rate*. A total of 4 studies investigated the mean HR response to heel stick [14, 26, 51, 65], while 1 study investigated the mean HR response to venipuncture [21]. All studies investigated infants between 3 and 7 postnatal days. Overall, mean HR was found to increase in response to acute pain. However, the magnitude of responses was variable and ranged from 154 to 183.4 bpm, with mean HR being higher in the study using venipuncture as the acutely painful stimulus. Additionally, variability in the magnitude of mean HR response may be due to the fact that over half of the studies are not including covariates in their analysis [14, 27, 65]. In the two studies that did include covariates, the authors controlled for the frequency of invasive procedures, severity of illness, ventilation status, sex, number of prior heel sticks, duration of blood draws, and baseline HR. Overall, the quality of the studies varied (i.e., 40% compared to 85%).

*Heart Rate Variability*. One high quality study investigated mean total HRV in response to heel stick at 5 postnatal days or less [14]. It was found that total HRV was not significantly different in response to heel stick (see Table 8). However, total HRV represented the standard deviation of the mean HR, which may have affected the accuracy of the measure.

*Late Preterm: 34 to 37 Weeks of GA*

*Mean Heart Rate*. A total of 2 studies investigated mean HR response to heel stick [24] or venipuncture [27]. The studies investigated infants at 3 to 7 postnatal days. In both studies, mean HR increased following the acute pain procedure. Mean HR in response to acute pain was found to be 165.3 and 163.2 bpm after heel stick and venipuncture, respectively. The quality of the studies was found to vary (i.e., 40% compared to 85%).

*Full Term: 37 to 42 Weeks of GA*

*Mean Heart Rate*. A total of 9 studies investigated mean HR response to heel stick [24, 38, 43, 45, 52, 55, 57, 63, 71], 4 in response to venipuncture [27, 34, 54, 66], and 1 in response to vaccination [51]. The studies investigated infants at 0 to 7 postnatal days. Overall, mean HR increased after acute pain procedure; however, as in premature infants, the magnitude of the response was variable in term born infants. Mean HR ranged from 134 to 174 bpm in response to acute pain. Out of the 14 studies investigating mean HR response to acute pain, only three included covariates in their analyses [38, 43, 57], which again may explain the variability in the results. These studies included the number of additional sticks required to obtain the blood sample, duration of the heel stick, frequency of crying, average HR, preintervention baseline (percentage of time crying in the last two minutes before beginning the interventions), breast-fed (yes/no), SSRI exposure (yes/no), age at time of acute pain, maternal analgesia (yes/no), dose of SSRI at time of delivery, and dose of clonazepam at time of delivery. Overall, the quality of the studies was variable and ranged from low to high quality.

*Maximum Heart Rate*. A total of 6 studies investigated maximum HR while infants underwent a heel stick procedure at 2 to 7 postnatal days [35, 36, 38, 41, 61, 68]. Overall, maximum HR was found to increase in response to the heel stick and ranged from 149 to 192 bpm (Table 9). Two studies included covariates in their analysis [38, 41], which were number of additional sticks required to obtain the blood sample, duration of the heel stick, frequency of crying, average HR, gestational age, birth weight, sex, mode of delivery, diabetic mother (yes/no), breast-fed one hour before puncture (yes/no), or received oral glucose (yes/no). Overall, the studies were relatively lower in quality (i.e., 60 to 75%).

*Heart Rate Change*. A total of 9 studies investigated mean HR change in response to heel stick [37, 45, 47, 48, 60, 61, 68, 70] and intramuscular injection [62] from 0 to 7 postnatal days. In all studies, mean HR increased significantly in response to acute pain. Mean HR was found to increase by 31 to 49 bpm or between 11 and 38 percent. Only three studies included covariates in their analysis of the cardiovascular [37, 61, 62], which included sex, nurse, number of lances needed, baseline HR, and activity. The studies included were generally high in quality.

*Heart Rate Variability*. A total of 8 studies investigated mean HF HRV, 6 studies investigated LF HRV, and 3 studies investigated the LF/HF ratio or total HRV during heel stick [42, 43, 46, 54, 57, 70, 71] or venipuncture [15]. Total HRV was found to be variable in the two studies, with one study suggesting its increase in response to heel stick [8] and the other study suggesting its decrease in response to venipuncture [15]. It is possible that the two acutely painful procedures may have differed in the amount of pain caused. There was also variability in HF HRV, with some studies finding HF HRV decreased in response to acute pain [42, 46, 57, 70, 71], and some studies finding no difference in HF HRV in response to acute pain [43, 54, 55]. These differences in response patterns may be due in part to the heterogeneity of covariates included in four of the studies [42, 43, 54, 55] and the lack of covariates included in the four remaining studies [54, 55, 70, 71]. LF HRV was found to decrease in response to acute pain in four studies [54, 57, 70, 71] and increase in one study [54]. Only one study included covariates in their analyses, which may help to explain the variability in the results [57]. Finally, the LF/HF ratio was found to increase in the three studies [57, 70, 71], with only one study including covariates in the analysis [57]. These studies included infants between 0 and 7 postnatal days, and the studies ranged in quality from 50 to 80%.

*Summary of Results: Age of Measurement Less Than 7 Postnatal Days*

*Mean Heart Rate*. The magnitude of cardiovascular response was variable across GAs, with those born at 25 to 27 weeks GA displaying a blunted HR response to acute pain, and those born at 28 to 42 weeks GA displaying an increase in HR across phases that increased in variability as GA increased.

*Mean Heart Rate Change*. Mean HR change was utilized in studies investigating infants born at 28 to 32 weeks and 37 to 42 weeks of GA. Both groups had a significant increase in HR following the acutely painful procedure. Maximum HR in response to a heel stick was utilized in infants' born at 37 to 42 weeks GA. All studies found that maximum HR increased in response to the heel stick.

*Heart Rate Variability*. Total HRV, LF HRV, HF HRV, and the LF/HF ratio were examined in infants born at 28 to 32, 32 to 34, and 37 to 42 weeks of GA. Although LF and HF HRV were found to increase and the LF/HF ratio decreased in response to acute pain in one study investigating those born at 28 to 32 weeks GA, clear patterns of HRV in response to acute pain could not be deciphered in the later born infants (i.e., 32 to 34 and 37 to 42 weeks of GA).

#### 3.2.2. Age at Measurement: One to Two Postnatal Weeks

No studies investigated cardiovascular responses to acute pain in extremely preterm infants in the first or second postnatal week of life.

*Very Preterm: 28 to 32 Weeks of GA*

*Mean HR Change*. One lower quality study investigated the mean change in HR following a heel stick at one postnatal week [44]. The authors found that infants' HR increased by approximately 5 to 10 bpm during the most invasive event of the blood collection. One study investigated mean HR change following a heel stick procedure in infants who were less than 14 postnatal days old [40]. During the procedure the authors found that mean HR increased significantly from baseline to heel stick procedure, with a mean HR change of 22.40 bpm and standard deviation of 15.42.

*Heart Rate Variability*. One high quality study investigated several components of HRV in response to a heel stick at 14 postnatal days or less [40]. The authors reported that LF and HF HRV increased in response to heel stick, while the LF/HF ratio decreased in response to heel stick. At the time of heel stick, mean LF HRV was reported at 69.84, with a standard deviation of 102.08, mean HF HRV was reported at 24.04, with a standard deviation of 40.90, and the LF/HF ratio was reported at 23.98, with a standard deviation of 21.39.

*Moderate to Late Preterm: 32 to 35 Weeks of GA*

*Mean Heart Rate*. One relatively high quality study investigated mean HR in response to a heel stick at less than 10 postnatal days [49]. Mean HR was found to increase in response to the heel stick and was approximately 159 bpm in response to the acute pain. A variety of covariates were included in the analysis of the cardiovascular measure, which included Apgar scores at 5 minutes, GA at birth, time since last painful procedure, number of painful procedures since admission, or received indomethacin in the past 12 hours (yes/no).

*Full Term: 37 to 42 Weeks of GA*

*Mean Heart Rate*. Two studies investigated mean HR response to heel stick [24] or venipuncture [67] at less than 15 postnatal days old. Mean HR was found to significantly increase in response to venipuncture and was reported at 163 bpm following the acutely painful procedure. In response to heel stick, mean HR was found to increase to 145.86 bpm with a standard deviation of 19.22 [24]. Overall, the quality of the studies was high.

*Summary of Results: Age of Measurement One to Two Postnatal Weeks*

*Mean Heart Rate*. Data were available from studies investigating those born at 28 to 32, 32 to 35, and 37 to 42 weeks of GA during the second postnatal week. Mean HR significantly increased in response to acutely painful procedures. The magnitude of HR responses was variable within and across GA groups.

*Heart Rate Variability*. One study investigated infants born at 28 to 32 weeks GA and found that they have an increased LF and HF HRV and a decreased LF/HF ratio in response to acute pain.

#### 3.2.3. Age at Measurement: Three Postnatal Weeks

No studies investigated cardiovascular responses to acute pain in very preterm, moderate to late preterm, or full term infants in the third postnatal week of life.

*Extremely Preterm: 24 to 26 Weeks of GA. *One high quality study investigated the mean and maximum HR response following a heel stick at 21 postnatal days [69]. The authors found that mean and maximum HR increased during the heel stick and were reported as 174.90 bpm with a standard deviation of 9.86 bpm and 175.91 bpm with a standard deviation of 10.35 bpm, respectively.

*Summary of Results: Age of Measurement at Three Postnatal Weeks*

*Mean Heart Rate*. In extremely preterm infants, mean and maximum HR were found to increase in response to acute pain at 3 postnatal weeks old. The blunted HR response that was noted in the first seven postnatal days was not found, suggesting an increased response to acute pain developing in extremely preterm infants in the first three weeks of life.

#### 3.2.4. Age at Measurement: One Postnatal Month

No studies investigated cardiovascular responses to acute pain in moderate to late preterm or full term infants in the third postnatal week of life.

*Extremely Preterm: 24 to 28 Weeks of GA*

*Mean Heart Rate*. Two studies with varying quality levels investigated mean HR following a heel stick procedure at four postnatal weeks [26, 59]. The authors found that there were significant increases in mean HR following the heel stick. The approximate mean HR response following the heel stick ranged from 170 to 190 bpm. A variety of covariates were included in one analysis [26], which comprised frequency of invasive procedures, severity of illness, ventilation status, and sex.

*Heart Rate Variability*. One study investigated LF and HF HRV and the LF/HF ratio during a heel lance procedure [59]. LF and HF HRV as well as the LF/HF ratio decreased during heel lance and were approximately 5.0, 1.0, and 8.0 during the heel lance, respectively.

*Very Preterm: 28 to 32 Weeks of GA*

*Mean Heart Rate*. One high quality study investigated mean HR following a heel stick at three to five postnatal weeks [56]. The authors found that mean HR was significantly higher during the heel stick than during recovery and was reported at 175.94 bpm, with a standard deviation of 12.66 bpm during the heel stick. The number of prior heel sticks, duration of blood draws, sex, and baseline HR were included as covariates in the analysis. 

*Summary of Results: Age of Measurement at One Postnatal Month*

*Mean Heart Rate*. Data from studies investigating infants at 24 to 28 and 28 to 32 weeks GA were available. Both studies found that mean HR increased in response to acute pain. Mean HR at one postnatal month was higher in response to acute pain, as compared to the first 7 postnatal days in those born at 28 to 32 weeks of GA.

*Heart Rate Variability*. At one postnatal month, one study found that LF and HF HRV and the LF/HF ratio decreased in response to acute pain in infants born at 24 to 28 weeks of GA.

#### 3.2.5. Age at Measurement: Two Postnatal Months

No studies investigated cardiovascular responses to acute pain in extremely, very, or moderate to late preterm infants in the second postnatal month of life.

*Full Term: 37 to 42 Weeks of GA*

*Mean Heart Rate*. One relatively lower quality study investigated mean HR responses to a heel stick procedure at two postnatal months [57]. The authors found that mean HR increased after heel stick and was approximately 190 bpm during the heel stick. The authors included a variety of covariates in their analysis (i.e., breast-fed (yes/no), SSRI exposure (yes/no), age at time of acute pain, maternal analgesia (yes/no), dose of SSRI at time of delivery, and dose of clonazepam at time of delivery).

*Heart Rate Variability*. The same study investigated mean HRV during the aforementioned acute pain procedure [57]. The authors found that, during the heel stick procedure, LF HRV and the LF/HF ratio decreased; however, there were no significant differences in HF HRV. HF and LF HRV and the LF/HF ratio were approximately 4.0, 28.0, and 8.0 during the heel stick procedure, respectively. The above-mentioned covariates were used in the analysis.

*Summary of Results: Two Postnatal Months*

*Mean Heart Rate*. One study investigated those born at 37 to 42 weeks GA. Mean HR was found to increase in response to acute pain.

*Heart Rate Variability*. Although LF HRV and the LF/HF ratio were found to decrease in response to pain, HF HRV was not significantly different from baseline to heel stick.

#### 3.2.6. Age at Measurement: Three Postnatal Months

No studies investigated cardiovascular responses to acute medical procedure pain in extremely preterm, very preterm, or moderate to late preterm infants in the third postnatal month of life.

*Full Term: 37 to 42 Weeks GA*

*Mean Heart Rate*. One relatively lower quality study investigated mean HR response following a heel stick at three postnatal months [53]. Mean HR increased after heel stick and was approximately 169 bpm during this time.

*Heart Rate Variability*. The same study investigated mean HRV during the aforementioned heel stick procedure [53]. In the study, the authors found that total HRV and the LF HRV increased during the heel stick; however there were no significant differences in HF HRV compared to baseline. When extrapolating the values, total HRV, HF, and LF HRV were approximately 4.10, 3.20, and 4.0 during the heel stick procedure, respectively.

*Summary of Results: Age of Measurement at Three Postnatal Months*

*Mean Heart Rate*. Data from one lower quality study investigating those born at 37 to 42 weeks GA were available. Mean HR was found to increase in response to acute pain.

*Heart Rate Variability.* Although total and LF HRV were found to increase in response to pain, HF HRV was not significantly different from baseline to heel stick.

#### 3.2.7. Age at Measurement: Four Postnatal Months

No studies investigated cardiovascular responses to acute pain in moderate to late preterm infants in the third postnatal week of life.

*Extremely Preterm: 24 to 28 Weeks of GA*

*Mean Heart Rate*. One relatively higher quality study investigated mean HR response following immunizations at four postnatal months [25]. The authors found that mean HR changed significantly across events, with significant increases from the end of baseline to first injection and from first injection to third injection. The approximate mean HR during the immunizations was 185 bpm. Corrected chronological age was included as a covariate in the analysis.

*Very Preterm: 28 to 32 Weeks of GA*

*Mean Heart Rate*. The same study investigated mean HR response following immunizations at four postnatal months in infants born at 29 to 32 weeks of GA [25]. The authors found that mean HR changed significantly across events, with significant increases from the end of baseline to first injection and from first injection to third injection. The approximate mean HR during the immunizations was 188 bpm. The above-mentioned covariate was included in the analysis.

*Full Term: 37 to 42 Weeks of GA*

*Mean Heart Rate*. One study investigated mean HR response following immunizations at four postnatal months in infants born at 38 to 41 weeks of GA [25]. The authors found that mean HR changed significantly across events, with significant increases from the end of baseline to first injection and from first injection to third injection. The approximate mean HR during the immunizations was 182 bpm. The above-mentioned covariate was included in the analysis.

*Summary of Results: Age of Measurement at Four Postnatal Months*. Only one cross-sectional, relatively higher quality study [25] investigated the effect of GA on mean HR response following immunizations. The authors found that mean HR increased in response to acute pain in all GA groups. However, there was no effect of GA group (i.e., 24–28, 29–32, and 38–41 weeks GA) on mean HR response.

## 4. Discussion

To our knowledge, this is the first systematic review investigating the development of cardiovascular indices of acute pain responding across the first year of life. Large gaps were elucidated in this review and suggest that the development of infant pain responding outside of the first month of life still remains largely unknown. By way of overview, when measuring HR in the first 7 days of life, the variability within each age group on these measures became larger as the infant's GA increased. Measures of HRV in the first 7 days of life seemed to show less variability within age categories as the child's GA increased. Data from other postnatal age groups (i.e., 2nd week, 3rd week, 1 month, 2 months, 3 months, and 4 months) were very sparse with patterns generally impossible to discern due to the total absence or presence of only 1 study.

The following paragraphs will discuss key findings and patterns in the results of the systematic review with specific attention to GA at birth, age at measurement, and type of cardiac measurement in response to acutely painful procedures. Limitations and review contributions to the literature, as well as key areas for future research based on the findings, will be highlighted.

### 4.1. Extremely Preterm

Those born at less than 28 weeks GA displayed a blunted HR response to acute pain in the first week of life. At three weeks, as well as one and four postnatal months, mean HR was found to significantly increase during acutely painful procedures, as compared to baseline HR. Mean HR was higher at four postnatal months than during the first postnatal month. This synthesis suggests that mean HR responses to acute pain may stabilize developmentally (i.e., to increase in response to a stressor as in older humans) in extremely preterm infants after the first postnatal week of life. At one postnatal month of life, LF and HF HRV and the LF/HF ratio decreased in response to acute pain.

Past research has noted a blunted pain response in the first week of life based on GA [74–76]. It is possible that the health status of the child at birth may affect the infants' ability to react to invasive procedures during the first week of life [77]. The increase in cardiac responding from the first to the fourth postnatal months of life suggests that extremely low GA infants begin to demonstrate increased physiological responses to acute pain as the cardiovascular system matures [74–76]. Although the relative quality of studies was good (76 to 86%), it is important to interpret this qualitative synthesis with caution, given the relatively small group of studies (*N* = 3) and the lack of covariates in two of the three studies.

### 4.2. Very Preterm

In infants born between 28 and less than 32 weeks GA, mean HR was found to significantly increase following an acutely painful procedure from birth to four months of age. Mean HR was found to be higher at four months compared to one to two postnatal weeks of life. As mentioned above with extremely preterm infants, this increase in mean HR is likely linked to an increase in the parasympathetic contribution to HR control [74–76].

Although HRV components were only investigated in one study of very preterm infants in the second postnatal week of life, LF and HF HRV were found to increase, while the LF/HF ratio decreased in response to acute pain. Caution should be taken when interpreting these HR and HRV results, as it is based on four studies (quality scores range from 62 to 85%) and a single study (quality score: 85%), respectively. Additionally, only one study included covariates in their analysis.

### 4.3. Moderate to Late Preterm

In infants born at 32 to less than 37 weeks GA, mean HR was found to increase in response to acute pain during the first postnatal week of life; however, the magnitude of responses was variable. Mean HR was found to be stable across the second week of life and increased in response to acute pain. The inconsistencies in mean HR may be due to differences in the acute pain procedure (i.e., heel stick versus venipuncture), the variability in quality of studies (40 to 85%), and the lack of covariates included in the analyses of more than half of the studies (3/5).

Additionally, when total HRV in response to acute pain was examined in the first week of life in those born at 32 to 34 weeks GA, it did not significantly differ from baseline HRV. This may be due to nonlinearity in heartbeats, which is necessary to measure HRV, being less apparent before 35 weeks GA [76]. Moreover, the conclusions are based on one study with adequate quality (75%), and there were no covariates included in the analysis.

### 4.4. Full Term

During the first four postnatal months of life, full term infants displayed an increase in mean HR in response to acute pain; however, the magnitude of responses was variable. A relative increase in mean HR over the first four postnatal months was noted and may reflect a developmental, relative increase in the parasympathetic contribution to HR control [75]. Conversely, the variability in results may be due to the following: the lack of studies per age group at two, three, and four postnatal months, the study authors including differing or no covariates in their analyses, and the variable quality of studies.

During the first postnatal week of life, total, LF, and HF HRV in response to acute pain were found to be inconsistent among full term infants. The LF/HF ratio was the only consistent measure of HRV, and it was found to decrease in response to acute pain across studies. At two postnatal months, LF HRV and the LF/HF ratio decreased in response to acute pain. At three months of age in response to acute pain, total and LF HRV increased. There were no differences in HF HRV when compared to baseline levels at two or three postnatal months.

As mentioned above, the inconsistency within the HRV domains may be explained by the linear statistics utilized by authors [76]. As well, only one study out of eight included covariates in their analysis, and the quality of the studies varied qualitatively (55 to 80%).

### 4.5. Limitations of This Review

It is possible that we have omitted relevant studies despite our detailed search strategy, and we specifically excluded non-English language studies. Additionally, group-specific data (i.e., age at measurement and GA) were separated based on available data and natural groupings, which on occasion led to overlap in GA groups.

With regard to analyzing HRV, studies differed on spectrum calculation methods and models of data analysis. Although terminology such as LF and HF bands is common in the field, studies differ on frequency limits of the bands. Other studies utilized linear statistical approaches of comparing means and variance, which has been reported as less sensitive in classifying HRV in infants [75].

Furthermore, it was difficult to draw conclusions across development and GA groups for cardiovascular responses to acute medical procedure pain, as the majority of studies did not include covariates in their analyses that could impact an infants' cardiovascular response to acute pain. It is important to keep in mind that the variability in mean HR and HRV components may be due to this lack of control within the studies.

### 4.6. Implications for Research and Clinical Practice

The presence of variability in HR in older preterm infants and full term infants presents an important clinical challenge to gold-standard measures such as the PIPP-R, N-PASS, COMFORT, and Bernese Pain Scale. For example, the PIPP-R has physiological items (i.e., HR) that are numerically scored on a four-point scale reflecting changes in each variable from baseline values [19]. Given that natural variability in HR responding exists across all GA groups, it is possible that infants with more naturally reactive heart rates, and not higher subjective pain, may have higher scores on this pain scale. This may lead to infants receiving unnecessary pharmacological pain relief. Future research and clinical practice should address this concern in order to provide appropriate pain management to these vulnerable infants.

A lack of control within the studies investigated has been highlighted, with only 13 out of 41 studies including covariates in their analyses. Moreover, the covariates utilized in the studies are divergent, which may have increased the amount of variability noted in the cardiovascular responses to acute pain. Future research in the area of infant pain should address this lack of control by identifying and controlling for factors that may affect an infants' cardiovascular response to acute pain in their own research. Examining the studies that did use covariates, key covariates that should seriously be considered for inclusion in all cardiac response to pain studies (depending on design) are gestational age, age at measurement (i.e., postnatal age, corrected chronological age), birth weight, time since last feeding, ventilation status, baseline (i.e., prehandling cardiac responding), length of painful procedure, number of painful procedures (e.g., how many draw attempts), illness severity, sex, and respiration rate.

## Supplementary Material

 Supplementary File 1 contains an example search strategy (i.e. Medline) that systematically paired terms related to acute pain procedures, cardiovascular measures, and infants (0-3 years of age). Supplementary File 2 is the quality checklist that was utilized to rate the studies that were included in our systematic review.

## Figures and Tables

**Table 1:** Study characteristics.

Study	*N*	Country	Gestational age	Postnatal age^*∗*^	Acute pain procedure	Cardiovascular measure	Study design	Quality score^∧^
Abad et al. [34]	15	Spain	37–42	<4 days	Venipuncture	Mean HR	CS; randomized trial	17
Altun-Köroğlu et al. [35]	25	Turkey	37–41	4–8 days	Heel stick	Maximum HR	CS; double-blind, placebo-controlled trial	13
Bilgen et al. [36]	34	Turkey	37–42	1–9 days	Heel stick	HR change (%)	CS; randomized trial	14
Bucher et al. [37]	20	Switzerland	37–41	4 days	Heel stick	HR change (bpm)	CS; randomized trial	14
Campos [38]	20	United States	37–42	2 days	Heel stick	Mean HR	CS; randomized trial	15
Cong et al. [40]	28	United States	28–32	<14 days	Heel stick	HR increase, HRV	CS; randomized cross-over trial	17
Cong et al. [39]	14	United States	30–32	<9 days	Heel stick	Mean HR, HRV	CS; randomized cross-over trial	17
Craig et al. [24]	56	Canada	25–27, 28–30, 31–33, 34–36, and 37–42	<8 days	Heel stick	Mean HR	CS; observational	17
de Jesus et al. [41]	41	Brazil	37–41	<2 days	Heel stick	HRV	CS; observational	15
de Oliveira et al. [42]	36	Brazil	37–41	<2 days	Heel stick	Maximum HR, HRV	CS; observational	14
Gormally et al. [43]	21	Canada	37–42	2 days	Heel stick	Mean HR, HRV	CS; randomized controlled trial	15
Goubet et al. [44]	14	United States	28–32	4 days, 21 days	Heel stick	HR change	C; observational	13
Gray et al. [45]	15	United States	37–42	<3 days	Heel stick	Mean HR	CS; randomized controlled trial	17
Greenberg [46]	21	United States	37–42	<1 day	Heel stick	Mean HRV	CS; randomized trial	13
Grunau et al. [25]	138	Canada	≤28, 29–32, and 38–41	4 months	Immunization	Mean HR	C; observational	16
Haouari et al. [47]	15	England	37–42	<6 days	Heel stick	HR change (%)	CS; double-blind, placebo controlled trial	15
Jatana et al. [48]	25	India	37–42	<7 days	Heel stick	HR change (bpm)	CS; randomized trial	10
Johnston et al. [49]	20	Canada	32–35	<10 days	Heel stick	Mean HR	CS; randomized cross-over trial	15
Johnston et al. [50]	89	Canada	27, 32	4 days, 5 weeks	Heel stick	Mean HR	CS; observational	15
Kostandy et al. [51]	19	United States	37–42	1 day	Hepatitis B vaccination	Mean HR	CS; randomized controlled trial	17
Leite et al. [52]	29	Brazil	37–42	<7 days	Heel stick	Mean HR	CS; randomized clinical trial	17
Lindh et al. [55]	25	Sweden	37–42	4-5 days	Heel stick	Mean HR, HRV	CS; observational	11
Lindh et al. [54]	28	Sweden	37–42	3 days	Venipuncture	Mean HR, HRV	CS; randomized, double-blind trial	15
Lindh et al. [53]	45	Sweden	37–42	3 months	DPT vaccination	Mean HR, HRV	CS; randomized, double-blind, controlled trial	14
Lucas-Thompson et al. [56]	49	United States	28–31, 32–34	3–5 days, 3–5 weeks	Heel stick	Mean HR	C; observational	17
Oberlander et al. [57]	23	Canada	37–42	2-3 days	Heel stick	Mean HR, HRV	CS; observational	13
Oberlander et al. [58]	22	Canada	37–42	2 months	Heel stick	Mean HR, HRV	C; observational	13
Oberlander et al. [59]	12	Canada	24–28	27–54 days	Heel stick	Mean HR, HRV	CS; observational	11
Ors et al. [60]	34	Turkey	37–42	<9 days	Heel stick	HR change (%)	CS; randomized trial	15
Owens and Todt [61]	20	United States	37–42	2 days	Heel stick	Mean HR	CS; observational	12
Sajedi et al. [62]	32	Iran	37–42	<1 day	Intramuscular injection	Mean HR	CS; randomized trial	13
Shibata et al. [63]	47	Japan	37–42	3-4 days	Heel stick	Mean HR	CS; observational	14
Singh et al. [27]	150	India	32–34, 35–37, and 37–42	<7 days	Heel stick	Mean HR	CS; observational	8
Stevens et al. [65]	40	Canada	32–34	<5 days	Heel stick	Mean HR	CS; descriptive	15
Stevens and Johnston [64]	124	Canada	32–34	≤5 days	Heel stick	Mean HR, maximum HR, and HRV	CS; observational	17
Taksande et al. [66]	80	India	37–42	<7 days	Venipuncture	Mean HR	CS; observational	11
Upadhyay et al. [67]	41	India	37–42	<15 days	Venipuncture	Mean HR	CS; randomized, placebo-controlled, double-blind trial	16
Uyan et al. [68]	21	Turkey	37–42	<11 days	Heel stick	HR change (%), maximum HR	CS; randomized controlled trial	14
Walden et al. [69]	11	United States	24–26 weeks	21 days	Heel stick	Mean HR, maximum HR	C; quasiexperimental, repeated measures	18
Weissman et al. [70]	29	Israel	37–42	2-3 days	Heel stick	HR increase (bpm), HRV	CS; randomized trial	11
Weissman et al. [71]	24	Israel	37–42	4–6 days	Heel stick	Mean HR, HRV	CS; randomized controlled trial	10

*Note.* CS = cross-sectional study, C = cohort study. CS and C quality scores are out of 20 and 21, respectively.

^*∗*^Postnatal age = age at measurement.

^∧^Out of 20 or 21 depending on research design.

**Table 2:** Description of study covariates included in the cardiovascular analyses.

Study	Covariates
Abad et al. [34]	N/A
Altun-Köroğlu et al. [35]	N/A
Bilgen et al. [36]	N/A
Bucher et al. [37]	Sex, nurse, number of lances needed, baseline heart rate, and activity
Campos [38]	The number of additional sticks required to obtain the blood sample, the duration of the heel stick, the frequency of crying, and the average HR
Cong et al. [40]	N/A
Cong et al. [39]	N/A
Craig et al. [24]	N/A
de Jesus et al. [41]	Gestational age, birth weight, sex, mode of delivery, diabetic mothers, breast-fed one hour before puncture, and received oral glucose
de Oliveira et al. [42]	PIPP score in the period before the heel prick
Gormally et al. [43]	Preintervention baseline (percentage of time crying in the last two minutes before beginning the interventions)
Goubet et al. [44]	N/A
Gray et al. [45]	N/A
Greenberg [46]	Age, weight, time since last feeding, heel stick and blood collection procedure length, and gestational age
Grunau et al. [25]	Corrected chronological age
Haouari et al. [47]	N/A
Jatana et al. [48]	N/A
Johnston et al. [49]	Apgar scores at 5 minutes, gestational age at birth, time since last painful procedure, number of painful procedures since admission, or received indomethacin in the past 12 hours
Johnston et al. [50]	Frequency of invasive procedures, severity of illness, ventilation status, and sex
Kostandy et al. [51]	N/A
Leite et al. [52]	N/A
Lindh et al. [55]	N/A
Lindh et al. [54]	N/A
Lindh et al. [53]	N/A
Lucas-Thompson et al. [56]	Number of prior heel sticks, duration of blood draws, sex, and baseline heart rate
Oberlander et al. [57]	Breast-fed, SSRI exposure, age at time of acute pain, maternal analgesia, dose of SSRI at delivery, and dose of clonazepam at time of delivery
Oberlander et al. [58]	Breast-fed, SSRI exposure, age at time of acute pain, maternal analgesia, dose of SSRI at delivery, and dose of clonazepam at time of delivery
Oberlander et al. [59]	N/A
Ors et al. [60]	N/A
Owens and Todt [61]	Sex
Sajedi et al. [62]	Sex
Shibata et al. [63]	N/A
Singh et al. [27]	N/A
Stevens et al. [65]	N/A
Stevens and Johnston [64]	N/A
Taksande et al. [66]	N/A
Upadhyay et al. [67]	N/A
Uyan et al. [68]	N/A
Walden et al. [69]	N/A
Weissman et al. [70]	N/A
Weissman et al. [71]	N/A

*Note*. N/A = not applicable.

**Table 3:** Mean and standard deviations for heart rate response to acute pain at less than 7 postnatal days.

Gestational age	Reference	Mean HR (bpm)	SD
25–27 weeks	Craig et al. [24]	172.38	17.22

28–32 weeks	Cong et al. [39]	165.00	14.00
	Craig et al. [24]	168.20	10.50
	Craig et al. [24]	155.25	21.57
	Lucas-Thompson et al. [56]	169.27	10.89

32–34 weeks	Singh et al. [27]	183.40	15.93
	Stevens and Johnston [64]	162.20	15.36
	Stevens et al. [65]	154.00	13.00
	Lucas-Thompson et al. [56]	158.18	15.19

34–37 weeks	Craig et al. [24]	163.20	27.82
	Singh et al. [27]	165.30	16.50

37–42 weeks	Abad et al.^*∗*^ [34]	170.00	N/A
	Craig et al. [24]	145.86	19.22
	Campos [38]	174.00	16.60
	Gormally et al^*∗*^. [43]	180.00	N/A
	Gray et al.^*∗*^ [45]	123.00	N/A
	Kostandy et al.^*∗*^ [51]	155.00	N/A
	Leite et al. [52]	172.70	21.50
	Lindh et al. [55]	134.00	19.00
	Lindh et al. [54]	144.00	20.00
	Oberlander et al.^*∗*^ [57]	168.00	N/A
	Shibata et al.^*∗*^ [63]	170.00	N/A

*Note*. *∗* denotes numbers that were extrapolated from graphs.

**Table 4 tab4:** Mean and standard deviations for heart rate change from baseline in response to acute pain at less than 7 postnatal days.

Gestational age	Reference	HR change	SD
28–32 weeks	Goubet et al.^*∗*^ [44]	0–15 bpm	N/A

37–42 weeks	Altun-Köroğlu et al. [35]	37.00%	N/A
	Bilgen et al. [36]	19.00%	N/A
	Bucher et al.^*∗*^ [37]	45 bpm	N/A
	Gray et al. [45]	36–38 bpm	N/A
	Haouari et al. [47]	11.40%	3.0
	Jatana et al. [48]	31.48 bpm	6.66 bpm
	Ors et al. [60]	19.00%	N/A
	Owens and Todt [61]	49.00 bpm	17.5 bpm
	Sajedi et al. [62]	10.81	N/A
	Uyan et al. [68]	38.20%	N/A
	Weissman et al. [70]	36.50 bpm	19.50 bpm

*Note*. *∗* denotes numbers that were extrapolated from graphs.

**Table 5 tab5:** Mean and standard deviations for low frequency heart rate variability in response to acute pain at less than 7 postnatal days.

Gestational age	Reference	Mean LF HRV	SD
28–32 weeks	Cong et al. [39]	17.62	24.55

37–42 weeks	Gormally et al.^*∗*^ [43]	1.65	N/A
	Lindh et al. [55]	4.2	0.4
	Lindh et al. [54]	4.00	0.39
	Oberlander et al.^*∗*^ [57]	11.0	N/A
	Weissman et al. [71]	1.45	0.38

*Note*. HRV = heart rate variability, LF = low frequency, SD = standard deviation, and *∗* denotes numbers that were extrapolated from graphs.

**Table 6 tab6:** Mean and standard deviations for high frequency heart rate variability in response to acute pain at less than 7 postnatal days.

Gestational age	Reference	Mean HF HRV	SD
28–32 weeks	Cong et al. [39]	23.52	35.96

37–42 weeks	de Oliveira et al. [42]	0.44	0.69
	Greenberg^*∗*^ [46]	2.5	N/A
	Lindh et al. [55]	3.4	0.60
	Lindh et al. [54]	3.23	0.45
	Oberlander et al.^*∗*^ [57]	2.0	N/A
	Weissman et al. [71]	0.76	0.50

*Note*. HRV = heart rate variability, HF = high frequency, SD = standard deviation, and *∗* denotes numbers that were extrapolated from graphs.

**Table 7 tab7:** Mean and standard deviations for low frequency/high frequency ratio in response to acute pain at less than 7 postnatal days.

Gestational age	Reference	Mean LF/HF ratio	SD
28–32 weeks	Cong et al. [39]	1.75	1.84

37–42 weeks	Oberlander et al.^*∗*^ [57]	6.00	N/A
	Weissman et al. [71]	6.1	3.2

*Note*. LF = low frequency, HF = high frequency, SD = standard deviation, and *∗* denotes numbers that were extrapolated from graphs.

**Table 8 tab8:** Mean and standard deviations for total heart rate variability in response to acute pain at less than 7 postnatal days.

Gestational age	Reference	Mean total HRV	SD
32–34 weeks	Stevens and Johnston [64]	4.52	2.95

37–42 weeks	Lindh et al. [55]	4.30	0.40
	Lindh et al. [54]	4.10	0.35

*Note*. HRV = heart rate variability, SD = standard deviation.

**Table 9:** Mean and standard deviations for maximum heart rate in response to acute pain at less than 7 postnatal days.

Gestational age	Reference	Maximum HR (bpm)	SD
37–42 weeks	Campos [38]	192.00	11.80
	de Jesus et al. [41]	149.00	N/A
	Owens and Todt [61]	179.40	13.40
	Singh et al. [27]	160.30	20.00
	Taksande et al. [66]	151.00	10.40
	Uyan et al. [68]	186.00	N/A

*Note*. bpm = beats per minute, HR = heart rate, SD = standard deviation.

## Additional Points

To our knowledge, this is the first systematic review of cardiac responding to acute pain within infancy. Forty-one studies were included to examine different methods of cardiac responses within developmentally sensitive categories. In response to acutely painful procedures, most infants had an increase in mean heart rate; however the magnitude of the increase showed great variability. Research in the area of heart rate variability was inconsistent limiting interpretation of studies using this measure. More attention to covariates and agreement on methodological factors related to cardiac measurement is needed to better understand this physiological response to pain.
