# Supplementary material for: Development of Cardiovascular Indices of Acute Pain Responding in Infants: A Systematic Review
Source: Pain Res Manag. 2016 Apr 20;2016:8458696. doi: 10.1155/2016/8458696 (PMC4904608; doi:10.1155/2016/8458696)
Supplement: Supplementary file 1 — contains an example search strategy (i.e. Medline) that systematically paired terms related to acute pain procedures, cardiovascular measures, and infants (0-3 years of age). Supplementary File 2 is the quality checklist that was utilized to rate the studies that were included in our systematic review. [file 8458696.f1.zip › Appendix 1_Medline Search Strategy.docx]

**Medline Search Strategy**

1. Needles/

2. needle*.mp.

3. (bloodsampl* or immuni* or inoculat* or vaccin* or inject* or "finger prick*" or finger-prick or "heel prick*" or heel-prick* or "heel lance*" or heel-lance* or "heel puncture*" or heel-puncture* or "heel stick" or suture* or (laceration* adj3 repair*)).mp.

4. ("lumbar puncture" or lumbar-puncture* or "spinal tap*" or spinal-tap*).mp.

5. ("bone marrow aspiration" or "bone marrow biops*").mp.

6. (intravenous or intra-venous or venepuncture* or venipuncture* or venous cannulation* or (arterial blood gas* and cannul*)).mp.

7. ((catheter adj6 insert*) or catheter* or port-a-cath* or portacath).mp.

8. ("central line" adj6 (insert* or remov*)).mp.

9. (central venous catheter* adj6 insert*).mp.

10. (localanalges* or local anaesthe* or local anesthe*).mp.

11. ((arterial puncture or artery) adj6 puncture*).mp.

12. "arterial line*".mp.

13. (thoracocentesis or paracentesis).mp.

14. 1 or 2 or 3 or 4 or 5 or 6 or 7 or 8 or 9 or 10 or 11 or 12 or 13

15. exp Pain/

16. Pain Measurement/

17. PAIN THRESHOLD/

18. pain*.mp.

19. 15 or 16 or 17 or 18

20. 14 and 19

21. ((vaccin* adj6 pain) or (cannul* adj6 pain) or (needle* adj6 pain*) or (procedure* adj6 pain*) or (procedure-related adj6 pain)).mp.

22. 20 or 21

23. Child, Preschool/

24. exp Infant/

25. (baby or babies or neonate* or newborn or child* or infant* or paediatric* or pediatric*).mp.

26. 23 or 24 or 25

27. 22 and 26

28. Heart Rate/

29. (physiology or physiological).mp.

30. heart rate variability.mp.

31. Psychophysics/

32. Autonomic Nervous System/

33. vagal tone.mp.

34. Electrocardiograph*.mp.

35. low frequency.mp.

36. high frequency.mp.

37. (biobehaviour or biobehavior).mp.

38. respiratory sinus arrhythmia.mp.

39. respirat*.mp.

40. (spectral analysis or spectrum analysis).mp.

41. (frequency domain measures or frequency domain analysis).mp.

42. Arterial Pressure/

43. 28 or 29 or 30 or 31 or 32 or 33 or 34 or 35 or 36 or 37 or 38 or 39 or 40 or 41 or 42

44. 27 and 43
