# Supplementary material for: Development of Cardiovascular Indices of Acute Pain Responding in Infants: A Systematic Review
Source: Pain Res Manag. 2016 Apr 20;2016:8458696. doi: 10.1155/2016/8458696 (PMC4904608; doi:10.1155/2016/8458696)
Supplement: Supplementary file 1 — contains an example search strategy (i.e. Medline) that systematically paired terms related to acute pain procedures, cardiovascular measures, and infants (0-3 years of age). Supplementary File 2 is the quality checklist that was utilized to rate the studies that were included in our systematic review. [file 8458696.f1.zip › Appendix 2-Quality Assessment Measure.docx]

**Appendix 2. Quality Assessment Measure**

| 1. Is the hypothesis/aim/objective of the study clearly described? |
| --- |
| 1. Are the main outcomes to be measured clearly described in the Introduction or Methods section? |
| 1. Is the design of the study described? |
| 1. Is the setting of the study described? |
| 1. Is the source of the subjects studied stated? |
| 1. Is the distribution of the study population by age described? |
| 1. Is the distribution of the study population by gender described? |
| 1. Is the sample size stated? |
| 1. Is the participation/follow up described? |
| 1. Are non-participants/subjects lost to follow up described? |
| 1. Are the main findings of the study clearly described? |
| 1. Are the statistical methods described? |
| 1. Have actual probability values been reported (e.g., 0.035 rather than < 0.05) for the main outcomes except where the probability value is less than 0.001? |
| 1. Are confidence intervals/standard deviations given? |
| 1. Are any conclusions stated? 2. Were the subjects asked to participate in the study representative of the entire population from which they were recruited? |
| 1. Were the subjects who were prepared to participate in the study representative of the entire population from which they were recruited? |
| 1. Was the participation/follow-up rate > 80%? |
| 1. Were the main outcome measures used accurate (valid and reliable)? |
| 1. Was the sample size justified? |
| 1. Analysis adjusts for length of follow up? |
